# Biological Mechanisms Underlying Structural Changes Induced by Colorectal Field Carcinogenesis Measured with Low-Coherence Enhanced Backscattering (LEBS) Spectroscopy

**DOI:** 10.1371/journal.pone.0057206

**Published:** 2013-02-19

**Authors:** Nikhil N. Mutyal, Andrew Radosevich, Ashish K. Tiwari, Yolanda Stypula, Ramesh Wali, Dhananjay Kunte, Hemant K. Roy, Vadim Backman

**Affiliations:** 1 Department of Biomedical Engineering, Northwestern University, Evanston, Illinois, United States of America; 2 Department of Internal Medicine, NorthShore University Healthsystems, Evanston, Illinois, United States of America; The Chinese University of Hong Kong, Hong Kong

## Abstract

We previously reported the utility of Low-Coherence Enhanced Backscattering (LEBS) Spectroscopy in detecting optical changes in uninvolved rectal mucosa, changes that are indicative of the presence of advanced colorectal adenomas elsewhere in the colon (field carcinogenesis). We hypothesized that the alterations in optical signatures are due to structural changes in colonocytes. To elucidate those colonocyte changes, we used LEBS and an early time point in an animal model of colorectal field carcinogenesis – rats treated with azoxymethane (AOM). Changes in LEBS markers in intact mucosa from AOM-treated rats could be at least partially attributed to changes in colonocytes. To investigate the molecular mechanisms underlying the colonocyte abnormalities in premalignant colon, we took a candidate approach. We compared expression profiles of genes implicated directly or indirectly in cytoskeletal dysregulation in colorectal tissues from saline-treated versus AOM-treated rats. Our data suggest that a number of genes known to affect colon tumorigenesis are up-regulated in colonocytes, and genes previously reported to be tumor suppressors in metastatic cancer are down-regulated in colonocytes, despite the colonocytes being histologically normal. To further understand the role of the cytoskeleton in generating changes in optical markers of cells, we used pharmacological disruption (using colchicine) of the cytoskeleton. We found that differences in optical markers (between AOM- and control-treated rats) were negated by the disruption, suggesting cytoskeletal involvement in the optical changes. These studies provide significant insights into the micro-architectural alterations in early colon carcinogenesis, and may enable optimization of both bio-photonic and molecular risk stratification techniques to personalize colorectal cancer screening.

## Introduction

Field carcinogenesis [1] is the notion that the genetic/environmental milieu that leads to a focal tumor exists not only at the tumor site, but can also be diffusely present throughout the organ. For instance, if a patient develops a colorectal cancer (CRC) at a particular location, the prevailing logic is that it occurred through interplay of both genetic and exogenous factors (diet, smoking, fecal stream mutagens, etc.) leading to stochastic mutational events [1]. Thus, the diffuse field changes provide a fertile mutational environment and hence a predisposition to carcinogenesis, while focal neoplastic lesions are being determined by stochastic mutations. It follows that these genetic/epigenetic perturbations can result in microscale and/or nanoscale alterations in structure in the histologically normal and uninvolved mucosa. For example, previous biomarker studies of the rectum, such as the number of aberrant crypt foci [2], epithelial proliferation and/or apoptosis [3]–[4], and alterations in gene expression or in protein profiles [5]–[7] each suggest that there are subtle alterations in the rectum when neoplasia is present elsewhere in the colon. This suggests the possibility of using the rectum as a surrogate site for probing the risk of CRC [8]–[11].

Our group has developed a novel breakthrough optical technology – low coherence enhanced backscattering (LEBS) – that allows detection of these micro-architectural manifestations of field carcinogenesis [8]–[11]. We adapted well-known EBS phenomena from physicists who used it to characterize the properties of materials. We modified it with the use of low-coherence light to characterize information at micro- and nano-scales (<300 nm) in tissue and cells in a way that is not possible by light microscopy [10]. The LEBS signatures are determined by the spatial variations of the tissue refractive index, which in turn are determined by the local concentrations of tissue constituents, the size and shape of scattering particles, and the inter-relationships of these particles within the cellular milieu [10], [12]–[13]. Thus, LEBS is capable of detecting changes in organelles (the cytoskeleton, ribosomes, chromatin, mitochondria, collagen fibrils etc.) that are known to be altered in early carcinogenesis in the histologically normal mucosa [14]–[15]. We validated our work with two independent animal model studies of colon carcinogenesis and a human ex-vivo rectal biopsy study [8]–[11], which indicated the capability of LEBS in identifying future risk of neoplasia. From a clinical and diagnostic perspective, the test performance characteristics of LEBS [AUC (89%), sensitivity (100%) & specificity (80%)] markers for detecting advanced lesions are excellent, which should make them widely acceptable as a pre-screening technique of choice [11].

LEBS interrogates the colonic mucosa with a spectrum of depths ranging from 50–300 µm [8]-[11]. Given the heterogeneity of the mucosa, there are numerous potential structures that LEBS could be detecting. These include epithelial cells (colonocytes), stromal cells (fibroblasts, inflammatory cells), and larger organized structures such as crypts or interstitial components of lamina propria. Given the well-established epithelial-stromal interactions during carcinogenesis, and the ability of both cells and lamina propria (collagen etc) to modulate light scattering, there is biological plausibility for these structures to be the origin of the aberrant LEBS signals in pre-malignant colon [16]–[19]. However, several lines of evidence suggest that within colorectal cells cytoskeletal alterations may be the drivers of the structural and hence LEBS changes [20]–[21]. Identifying the origin of these changes is of importance not just from a cancer biology perspective, but also, to guide the design of LEBS fiber-optic probes for *in vivo* use in order to maximize diagnostic performance of LEBS markers. To determine the origin of the LEBS pre-carcinogenic signal, we studied AOM-treated rat model using the differential (effect size and delta Δ) between AOM-treated and saline-treated animals as our read out. We independently studied single cell preparations (predominantly colonocytes) versus intact tissue structures to assess the biological origin of aberrant LEBS signals. We now provide preliminary insights into the mechanism underlying the LEBS signal by demonstrating that there are indeed cytoskeletal alterations in field carcinogenesis. This is suggested by our observation that pharmacological disruption of the cytoskeleton diminished the Δ and effect size between AOM- and saline-treated animals. These studies provide the first demonstration that LEBS changes originate, at least partially, from cytoskeletal alterations in histologically normal, premalignant colon.

## Materials and Methods

### Low-Coherence Enhanced Backscattering (LEBS) Spectroscopy

**Figure 1:**
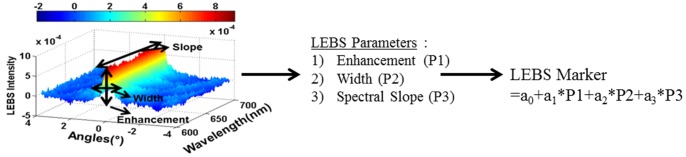
Representative image of LEBS peak from intact rat tissue. LEBS images show the different parameters (E, W & S) calculated from this peak and the scheme for calculation of LEBS marker using logistic regression of LEBS parameters by linear combination.

Our LEBS setup is described elsewhere [8]–[10]. All measurements were done using a LEBS instrument that enables simultaneous measurement of light scattering for a spectrum of wavelengths (400 – 700 nm) and for a range of backscattering angles (-15° to +15°). The angular measurements were used to identify an enhanced backscattering peak and then the spectral properties of the enhanced backscattering were measured. The LEBS peak (Fig. 1) can be characterized by three parameters. (1) LEBS width (W) was defined as the average full width at half maximum of an LEBS peak in the wavelength range of 540 to 640 nm. (2) A LEBS enhancement factor, (E), was the average height of the LEBS peak over this same wavelength range. (3) The LEBS spectral slope, (S), was calculated as the linear coefficient B from a linear regression of the form I_LEBS_ (λ)  =  A-Bλ, where I_LEBS_ (λ) is the intensity at the LEBS peak for wavelength λ [11].

### Statistical Analysis

The combined logistic regression for a LEBS marker is calculated using the scheme outlined previously [11]. In short, a binary logistic regression was used to build a multivariable model to predict AOM treatment (yes = 1, no = 0) from analysis of intact tissue. Three LEBS parameters (E(P_1_), W(P_2_) & S(P_3_)) were used as predictors by doing uni-variate analysis (ANOVA). To statistically construct a multivariable logistic model, all parameters with p<0.25 (all three parameters) from univariate logistic regression were entered into the model and removed backwardly. The final model retains parameters with p<0.05. To reduce concerns about the lack of robustness generated from a model derived from correlated predictors, the correlation coefficient was calculated for the selected parameters and verified to be non-significant. The final combined LEBS marker was built as a linear combination of LEBS parameters as follows: LEBS Marker  =  (a_0_) + (a_1_*P_1_) + (a_2_*P_2_) + (a_3_*P_3_) . The coefficients (a_i_’s) were empirically determined to maximize the separation between control and precancerous intact tissues. Prediction rule development was done using intact tissue and then applied to colonocytes with and without AOM treatment. All p-values were calculated using the built-in standard Student’s tests and are two sided with unequal variance in Excel (Microsoft Corp., Redmond WA). The effect size parameter (difference) between two groups was calculated as
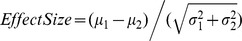



where μ_1 &_ μ_2_ are the means for the precancerous and control groups respectively and σ_1 &_ σ_2_ are the corresponding standard deviations. The delta parameter (Δ) was calculated by subtracting the average value of the LEBS marker (µ_1_–µ_2_) for the control from a precancerous sample.

### Animal Studies

**Figure 2:**
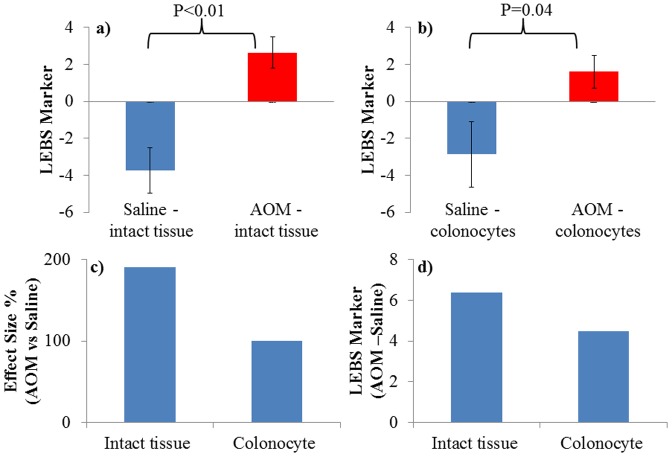
Structural Changes from intact colon tissue are partially from colon cells. a,b) The LEBS marker is altered in the intact AOM-treated rat tissue and in isolated colonocytes as compared to saline-treated rats, demonstrating differences in micro- and nano-architecture that can be captured with the LEBS technique. The magnitude of the alteration in the LEBS marker is higher for intact tissue compared to colonocytes. **c,d)** The effect size (2.c.) and delta (2.d.) between AOM-treated and saline-treated rats are higher for intact tissue than for isolated colonocytes. Because colonocytes are part of the intact tissue, this result suggests that colonocytes are partially responsible for differences in LEBS marker from intact tissue.

All animal procedures were reviewed and approved by the Institutional Animal Care and Use Committee for Northshore University Healthsystems. Thirty six Fisher 344 rats (150–200 g; Harlan, Indianapolis, IN) were randomly divided into two groups of 18 rats each and treated with two weekly injections (i.p.) of 15 mg/kg AOM (Midwest Research Institute, Kansas City, MO) or saline. Rats were euthanized 10 weeks after the second AOM injection and necropsy was done to rule out the presence of any adenomas in the colon after opening it longitudinally. The 10-week time point was chosen because it represents an early precancerous stage – there were no biochemical or cytological markers to characterize the tissue as pre-cancerous [22]. At least nine independent readings (sites) were taken from intact colon tissue of every rat (Fig. 2 a, c, d).

### PCR Arrays for Cytoskeletal Genes

**Figure 3:**
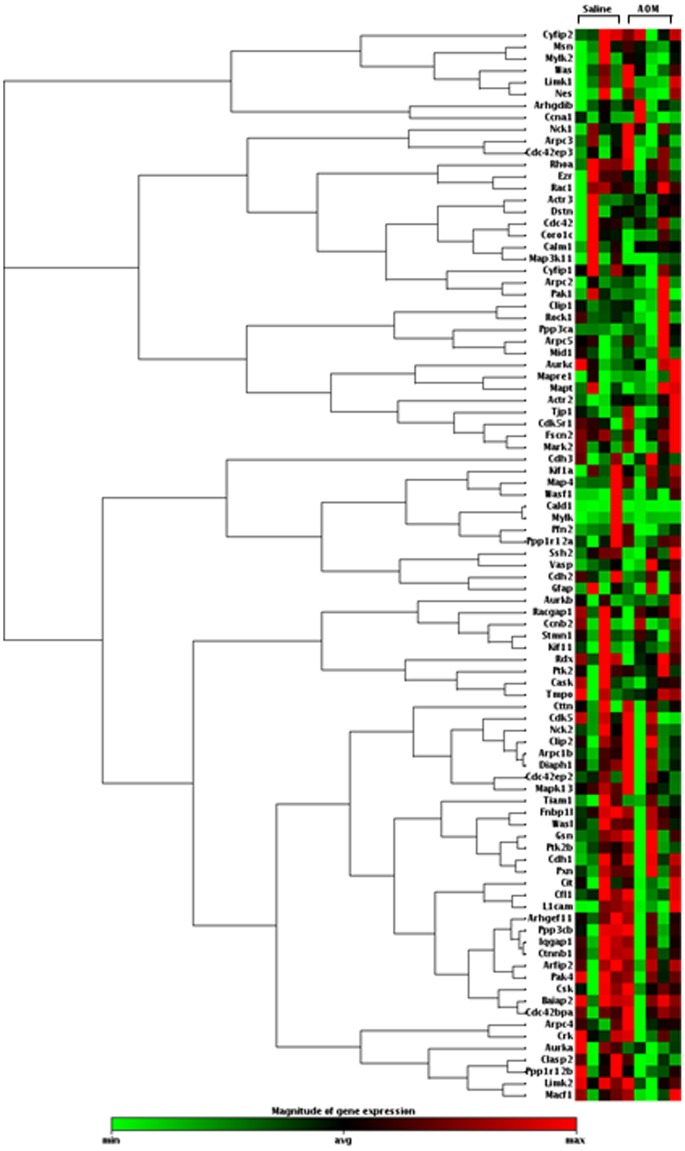
Clustergram for 9 rats (four saline and five AOM) showing the fold up- or down-regulation of genes involved in cytoskeleton regulation. The alterations in cytoskeletal gene regulation of precancerous (AOM-treated) rat cells compared to control (saline-treated) rat cells suggest possible involvement of the cytoskeleton in determining the structural differences measured by LEBS (Fig. 2).

A rat cytoskeleton regulator PCR assay (SA Biosciences, Frederick, MD) was done on colonocytes obtained from rats injected with saline (control) or azoxymethane (AOM) (Fig. 3). RNA from distal sections of the colon was isolated from 9 age-matched animals (4 in control and 5 in AOM group) using TRI Reagent (Molecular Research Center Inc, Cincinnati, OH), following a standard protocol for RNA isolation. Technical replicates were done for all nine samples from each group to verify reproducibility. Average fold differences in gene expression were calculated individually for each assay and then averaged for each animal group using the comparative Ct method after removing outliers. The threshold of fold change significance was set as >1.5 (up-regulation) and <0.67 (downregulation). The differential expression of the candidate genes in the AOM rat model was independently confirmed using individual TaqMan gene expression assays by real time PCR, according to the manufacturer’s instructions (Life Technologies, Grand Island, NY).

### Isolating Colonocytes from Rat Colon

**Figure 4:**
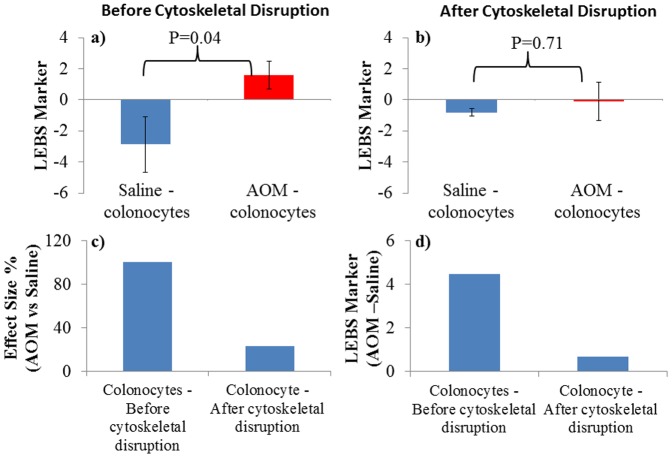
Structural Changes from colon cells are primarily from aberrant cytoskeleton. **a,b)** The graphs show the difference in LEBS markers for colonocytes from Saline-treated and AOM-treated rats, before and after cytoskeletal disruption. AOM treatment significantly altered (p<0.05) the LEBS marker compared to saline-treated controls. Disruption of the cytoskeletal assembly with colchicine nullified this difference (p = 0.71) **c,d)** The effect size and delta between LEBS markers from saline-treated and AOM-treated rat colonocytes are higher before cytoskeletal disruption. The difference in structure measured by the LEBS marker between saline-treated and AOM-treated rat colonocytes was negated by disruption of the cytoskeleton, suggesting that the cytoskeleton plays a role in determining or signaling intracellular changes in early carcinogenesis.

The colon was cleansed of all fecal matter by flushing with wash buffer {1 mM DTT (Dithiothreitol) in normal saline}. The colonocytes were harvested using a technique that combined chelation of divalent cations and mild mechanical dissociation as described previously [23]. The distal end of the colon was marked and clamped. The entire colon was then filled with filling buffer (0.5 mM DTT and 1.5 mM EDTA in normal saline) through the proximal end. The buffer-filled colon was incubated in normal saline at 37 degrees Celsius for 30 minutes with gentle rocking. At the end of the incubation, colonocytes were collected from the distal end of the colon through gentle squeezing and collected in 15 mL falcon tubes. The colonocyte-containing tubes were centrifuged for 5 minutes at 900 rpm at 4°C to form a solid cell pellet, and the supernatant was carefully discarded. Colonocytes were transported on ice in 5 ml of transparent RPMI media (GIBCO) containing protease inhibitors to prevent degradation. For the experiment (Figs. 2 & 4), at least nine readings were taken from 18 pellets placed in a circular glass chamber slide from the distal colon of saline-treated rats and AOM-treated rats.

### Colchicine treatment

For rat colonocytes, the tube containing the pellet was treated with 10 µM Colchicine (Sigma-Aldrich, St. Louis, MO) for 30 minutes. The protocol outlined in [24] was used to evaluate the effect of toxicity of the drug on the colonocytes and find the minimal dose sufficient to induce cytoskeletal disruption without causing microscopic abnormalities in cells. The dose was determined to be 10 µM for 1 ml of colonocyte pellet solution [25]. By verifying similar morphology using microscopy (data not shown), we verified that in colonocytes for both groups only cytoskeletal assembly was disrupted by treatment with colchicine. LEBS measurements were then obtained in a way similar to that described above. The variability in optical properties caused by the length of incubation with colchicine was estimated to be negligible (<3%) up to 30 min; nevertheless, the incubation time was tightly controlled at 30 min.

## Results and Discussion

### Micro-architectural changes observed in intact colon tissue are partly due to structural changes in cells

We did LEBS analysis on intact colon tissues as well as on a colonocyte pellet obtained from saline-treated (control) and AOM-treated (pre-cancerous) rats. The LEBS marker, which measures structural changes, was confirmed (Fig. 2a) to be significantly (P<0.01) increased for precancerous tissue compared to control intact tissue as demonstrated in our earlier studies [8]–[9]. Interestingly, the same LEBS marker was increased in precancerous colonocytes compared with controls (P = 0.04 Fig. 2b) albeit the magnitude of the difference was smaller. This observation was confirmed by analysis of the effect size (Fig. 2c) and delta (difference in average LEBS marker for pre-cancerous and control groups Fig. 2d). The effect size between precancerous and control group (Fig. 2.c.) was greatest (192%) for intact tissue that included structural changes captured from colonocytes, stroma and crypts. The effect size was smaller (100%) (but still statistically significant) in isolated colonocytes compared to intact tissue (Fig. 2.d.) since only the structural changes within colonocytes were captured here by the LEBS marker. These results show that micro-architectural changes measured in intact tissues by LEBS markers receive a major contribution from structural changes in colonocytes, but also include contributions from other structures.

### Subcellular structural differences are partially due to intra-cellular cytoskeletal organization

We hypothesized that changes in optical properties from colonocytes as measured by LEBS are due to alterations in the nano-scale and micro-scale structures of cells during early carcinogenesis. Since the cytoskeleton is a key building block of the cell, it is likely that cytoskeletal organization contributes to changes in optical properties. To investigate this hypothesis, we did a cytoskeletal PCR array for 92 genes. The cytoskeleton regulators PCR array revealed several candidate genes that were either significantly up-regulated or significantly down-regulated during early colorectal carcinogenesis in the AOM-treated rat model. A representative heat map from a single PCR assay showed changes in gene expression for the 92 cytoskeleton regulators (Fig. 3). Upon averaging fold changes in the animals, genes that were up-regulated in the AOM group included Cdk5r1, Limk1, Mapt, and Was. All of these genes regulate metastatic potential in various cancers. For example, Limk1 plays an important role in cell cycle progression and knockout of this gene inhibits metastatic behavior [26]. Genes that were down-regulated included Aurka, Macf1, and Pfn2. The gene Pfn2 codes for the Profilin 2 protein, a member of the profilin family, which has previously been described as a tumor suppressor in metastatic cancers [27]. We independently verified the modulation of these candidate genes in the AOM rat model using individual TaqMan gene expression assays, thus, confirming the results of the PCR array (data not shown). We considered the modulation of cytoskeletal genes as a factor that may be responsible for changes in cytoskeletal organization and in turn may contribute to changes in the optical properties of cells. This alteration in cytoskeletal genes prompted us to investigate the role of the cytoskeleton in causing the differences in optical properties between normal and premalignant cells.

### Disruption of cytoskeletal organization by a pharmacological agent negates the differences in subcellular morphology

To test the general hypothesis that cytoskeletal organization is involved in determining the changes in tissue micro-architecture measured by LEBS markers, we treated the colonocyte pellet with colchicine. Colchicine is a pharmacological agent that selectively inhibits microtubule polymerization (due to its specific binding with tubulin), stops cell division, and inhibits intra- and inter-cellular communication [24]. We treated rat colonocytes with a minimum concentration and duration of colchicine in order to avoid any confounding effects and to achieve microscopically comparable populations of cells with minimal changes in their morphology. We tested the specific hypothesis that after treatment with colchicine, the difference in optical properties (as measured by LEBS markers) between the control and precancerous cells will be nullified due to the disruption of the cytoskeletal structure. It can be seen that the difference in LEBS markers (Figs. 4 a, b) after treatment with colchicine became statistically insignificant (p  =  0.71) between control and precancerous colonocytes. Reductions in effect size and delta (Figs. 4 c & d) between control and precancerous colonocyte pellet after treatment with colchicine became statistically insignificant, which also supported our hypothesis. This indicates that the difference between control and precancerous cells measured by LEBS markers are dependent on maintenance of the structural integrity of and differences in the cytoskeleton.

### Discussion

The purpose of this study was to understand the biological origins of the altered LEBS signal detected in colorectal field carcinogenesis. LEBS has the ability to detect changes in tissue structures with sensitivity to length scales from 100–800 nm. The LEBS signal can be affected not only by intra-cellular changes, but also by changes in the local microenvironment (e.g., the organization of crypts) [14]–[18]. Also, stromal alteration may induce profound optical changes reflecting aberrant epithelial-stromal interactions during carcinogenesis [19]. Our data suggest that LEBS detects both cellular and extracellular changes (Fig. 2). The cellular component may be related to expression of cytoskeletal genes because we found that there are profound alterations in cytoskeletal gene expression in the premalignant mucosa of AOM-treated rats (Fig. 3). That cytoskeletal changes contribute to changes in the LEBS signal was supported by our finding that pharmacological disruption of the cytoskeleton by colchicine negates the observable difference in LEBS between control and precancerous cells (Fig. 4).

The mucosa in colorectal field carcinogenesis is widely thought to be microscopically normal [1]. However, recent studies of colorectal field carcinogenesis have demonstrated that there are subtle structural differences in tissue components of the mucosa [2]–[7]. Thus, there are two possible mechanisms behind altered LEBS signals: (1) subtle differences in microscopically detectable structures that require rigorous quantitation to detect; (2) changes in structures whose size is below the sub-diffraction limit of light, which cannot be detected by standard light microscopy. With regards to the first mechanism, there is some evidence that crypt length, if rigorously quantified, is different in the uninvolved rectal mucosa of patients harboring neoplasia when compared to those that are neoplasia-free [14]. Our preliminary data for the AOM-treated rat model supports the first mechanism [28]. The second mechanism involves changes in structures below the limit of resolution of light microscopy (∼300–500 nm). Analysis by standard light microscopy will be insensitive to changes in mitochondria, ribosomes, high order chromatin structures and other organelles that have been implicated in early carcinogenesis. Since, LEBS is a quantitative signal it may be affected by both these mechanisms, and thus may be altered by subtle crypt changes and can also sense sub-diffraction structures that are >50 nm.

Changes in the cytoskeleton are an integral part of colorectal carcinogenesis. For instance, mutation of the *adenomatous polyposis coli* (APC) gene is the initiating event in greater than three-quarters of all CRCs, and the APC protein is a key regulator of microtubules [29]. Moreover, there have been striking changes seen in the cytoskeleton of microscopically normal cells from patients with familial adenomatous polyposis – patients who have a germline *APC* mutation [30]. Furthermore, many other proteins that are aberrant in early colon cancer (e.g. c-src, E-cadherin, β-catenin etc) also interact with the cytoskeleton. Thus, changes in cytoskeletal organization in premalignant tissue may contribute to aberrant protein trafficking, mitosis, etc. However, few if any reports show cytoskeletal proteins being altered in the microscopically normal mucosa. To the best of our knowledge, the only major report is one involving proteomic analysis of the pro-carcinogenic secondary bile acid [4].

Therefore, to do a preliminary evaluation of the role of the cytoskeleton in early colon carcinogenesis, and to provide a basis for further exploration of the role of the cytoskeleton in precancerous intracellular changes, we did cytoskeletal gene PCR arrays on colonic tissue from AOM-treated rats. We found up- and down-regulation of a number of cytoskeletal genes. Although, the experiments had only a modest sample size (n = 9 and n = 18 with technical replicates) and we analyzed for an extensive number of cytoskeleton-related genes (n = 92). Our results are consistent with early dysregulation of the cytoskeleton in field carcinogenesis and are in agreement with previous studies [31] on altered gene expression profiles in the AOM-treated rat model. For instance, it was observed that in early carcinogenesis (10–20 weeks after the 2^nd^ AOM injection), β-catenin was altered in 100% of dysplastic aberrant crypt foci (ACF’s). Also, β-catenin is known to anchor the actin cytoskeleton and is responsible for transmitting the contact inhibition signal that causes cells to stop dividing.

In order to demonstrate that these cytoskeletal proteins may be important in the modulation of LEBS signal that has been detectable in field carcinogenesis, we took the approach of disrupting micro tubular network with colchicine. We reasoned that if cytoskeleton was integral to the altered LEBS signals, then colchicine treatment should ameliorate the LEBS differences between the LEBS marker from the saline versus AOM-treated rat colonocytes. Although our experimental data (Fig. 4) using colchicine were consistent with a role for cytoskeletal changes in the alteration in the LEBS signal in field carcinogenesis, there are a number of caveats. First, the cytoskeleton is complex whereas colchicine is a selective inhibitor of microtubule formation. Second, pharmacological agents may have non-specific targets as well. Third, dose and timing were not optimized, and colonocytes exposed to colchicine may not be viable for a protracted time period. Despite these concerns, our cytoskeleton disruption experiments powerfully bolster the notion that cytoskeleton is one of the drivers behind LEBS marker alterations in field carcinogenesis. Although we demonstrated that the cytoskeleton plays a central role in determining the structural difference between premalignant and control phenotypes, there may be other independent or correlated mechanisms which could also contribute to differences in structure, a possibility that requires further study.

Our group previously reported similar changes in tissue micro-architecture measured by LEBS markers in early carcinogenesis [8]–[11]. In those studies, LEBS markers were obtained from intact ex vivo human rectal tissue and from animal models. This indicates that it is possible to measure structural manifestations of early carcinogenesis in human biopsies. In our earlier studies [8]–[11], we reported that LEBS markers in rectal tissue increased with progression of carcinogenesis. Studies of pellets of isolated colonocytes (Fig. 2 a, b) indicate a similar change in the marker when cells are tested. The fact that tissue architecture is no longer present in such pellets implies an intracellular origin of structural abnormalities in early carcinogenesis. Although we carried out the study in AOM-treated rats, the mechanisms underlying the biological origin of the structural changes can be extrapolated to humans because the AOM-treated rat model has a close resemblance to the morphological, genetic and cellular events in human colon carcinogenesis and to a well-defined chronology of genetic mutations and lesions [22]. For example, there are similar increases in proliferative indices in the 8–10 week-old AOM-treated rat and in the endoscopically normal mucosa of patients harboring adenomas. Thus the 8–10 week time point of the AOM-treated rat should correlate well with the uninvolved colonic mucosa of patients at elevated risk for developing neoplasia [22]–[23]. Therefore, we believe that part of our LEBS signal from premalignant rectal tissue is from the colonocytes, and that the difference between control and precancerous colonocytes can mostly be attributed to cytoskeletal alterations in early carcinogenesis. This conclusion was also supported by a recent study [32] by our group with an optical technique that is capable of looking at single cell nanostructure changes. It was demonstrated that the disorder strength parameter for rectal colonocytes (obtained by brushing) are capable of risk stratifying patients for colon cancer based on field carcinogenesis [32].

Although in this manuscript we provide evidence that colonocytes partially contribute to structural differences in intact tissue measured by LEBS marker, there might be limitations to this study. First, although the process of colonocyte isolation and pelleting is well established and validated [20], it could have an effect on the structural integrity of the cell. For instance, cells in rectal mucosa have particular directionality (polarity) and lose it when they are pelleted and form a homogenous blob. However, this effect, if present, should be similar between control and precancerous (AOM) colonocytes since their protocol for isolation and preparation is same. Therefore we do not believe that this potential confound could generate the observed differences in LEBS markers between AOM and control colonocytes. To further assuage this concern, we ensured that technical procedures such as colonocyte isolation and cell pelleting did not increase variability in optical properties by calculating the standard deviation of the average LEBS markers for different sets of cell pellets within each group and found it to be negligible (<8%) relative to the size of the difference between control and precancerous cells (∼100%). Furthermore, pellet to pellet variability (6%) for different preparations turned out to be statistically non-significant (p > 0.63). A similar concern can be raised for the colchicine experiment. However, we made sure that we used minimal colchicine concentrations and that the incubation periods were kept under tight control for both control and precancerous colonocytes. Use of this minimal concentration caused cytoskeletal disruption but kept the cellular morphology the same (observed under light microscopy) ensuring that changes observed in the LEBS marker originated solely from changes in cytoskeletal integrity. The changes in LEBS parameters (E, W&S) provide valuable physical insights into ultrastructural properties of tissues, but this is beyond the scope of the current study.

### Conclusion

We demonstrated that the micro- and nano-architectural changes observed in colorectal field carcinogenesis can partially be attributed to sub-cellular structural changes in colonocytes. Other micro/nano-architectural changes in intact tissue may possibly originate from the cellular microenvironment (cryptal organization, extracellular matrix). Using colonocytes extracted from AOM-treated rat colon we established that LEBS is sensitive to sub-cellular organization and can distinguish precancerous from normal cells. We demonstrated that the cytoskeleton plays a pivotal role in determining cellular differences in LEBS markers and hence in our ability to differentiate between control and precancerous cells. The ability of LEBS to detect early structural changes in field carcinogenesis will likely have application as an early cancer detection technique.
